# Epidemiologically Linked COVID-19 Outbreaks at a Youth Camp and Men’s Conference — Illinois, June–July 2021

**DOI:** 10.15585/mmwr.mm7035e4

**Published:** 2021-09-03

**Authors:** James Matthias, Sarah Patrick, Ann Wiringa, Amanda Pullman, Stephanie Hinton, Jon Campos, Terri Belville, Mallory Sinner, MPH, Torrie T. Buchanan, Bryan Sim, Kristin E. Goldesberry

**Affiliations:** ^1^CDC COVID-19 Response Team; ^2^Illinois Department of Public Health; ^3^Adams County Health Department, Quincy, Illinois; ^4^Schuyler County Health Department, Rushville, Illinois; ^5^Illinois Department of Public Health Laboratory, Springfield, Illinois.

On June 30, 2021, the Illinois Department of Public Health (IDPH) contacted CDC concerning COVID-19 outbreaks at two events sponsored by the same organization: a 5-day overnight church camp for persons aged 14–18 years and a 2-day men’s conference. Neither COVID-19 vaccination nor COVID-19 testing was required before either event. As of August 13, a total of 180 confirmed and probable cases had been identified among attendees at the two events and their close contacts. Among the 122 cases associated with the camp or the conference (primary cases), 18 were in persons who were fully vaccinated, with 38 close contacts. Eight of these 38 close contacts subsequently became infected with SARS-CoV-2, the virus that causes COVID-19 (secondary cases); among the eight close contacts with secondary cases, one half (four) were fully vaccinated. Among the 180 total persons with outbreak-associated cases, five (2.8%) were hospitalized; no deaths occurred. None of the vaccinated persons with cases were hospitalized. Approximately 1,000 persons across at least four states were exposed to SARS-CoV-2 through attendance at these events or through close contact with a person who had a primary case. This investigation underscores the impact of secondary SARS-CoV-2 transmission during large events, such as camps and conferences, when COVID-19 prevention strategies are not implemented. In Los Angeles County, California, during July 2021, when the SARS-CoV-2 B.1.617.2 (Delta) variant was predominant, unvaccinated residents were five times more likely to be infected and 29 times more likely to be hospitalized from infection than were vaccinated residents (*1*). Implementation of multiple prevention strategies, including vaccination and nonpharmaceutical interventions such as masking, physical distancing, and screening testing, are critical to preventing SARS-CoV-2 transmission and serious complications from COVID-19.

## Investigation and Findings

The camp was held during June 13–17, 2021, and included persons aged 14–18 years from a church organization with multiple locations across western Illinois, Iowa, and Missouri. A total of 294 campers arrived on buses or large passenger vans and were met by 41 staff members. No proof of COVID-19 vaccination or SARS-CoV-2 pretesting or testing on arrival was required, and the list of suggested items to bring to camp did not include masks. Campers were housed in large, shared boarding facilities of approximately 100 campers each, dined in a cafeteria together, participated in indoor and outdoor small group activities in which campers were with the same persons during program events, and participated in activities with all campers during all 5 days.

On June 16, the second to last camp day, one camper departed after becoming ill with a fever and respiratory symptoms and subsequently received a laboratory-confirmed diagnosis of COVID-19. Campers and staff members were notified, encouraged to receive SARS-CoV-2 testing, and instructed to quarantine per CDC guidance and isolate if they received a positive test result.*

Six camp staff members who received positive SARS-CoV-2 test results also attended the conference during June 18–19 but did not receive their results until after the conference ended; all six staff members had symptom onset during June 17–29.^†^ The conference was held at a different location from the camp and included 500 attendees and 30 staff members, and, as with the camp, no COVID-19 vaccination, SARS-CoV-2 testing, or masking was required. The first case in a conference attendee was diagnosed on June 21, 2 days after the conference. After conference-associated COVID-19 cases were identified, conference attendees and staff members were notified, encouraged to receiving SARS-CoV-2 testing, and instructed to quarantine per CDC guidance and isolate if they received a positive test result.

A confirmed case was defined as receipt of a positive SARS-CoV-2 nucleic acid amplification test result in a camp or conference attendee, and a probable case was defined as receipt of a positive SARS-CoV-2 antigen test result.^§^ Cases were identified through case investigation after laboratory notification of a positive test result. Information on symptom onset or specimen collection dates (available for 174 [97%] of 180 persons), COVID-19 vaccination status (from the state immunization registry), county of residence, test results, and viral sequencing data (available for 31 [17%] persons) was collected for persons with camp- and conference-associated cases (primary cases). IDPH’s contact tracing system identified close contacts of persons with primary cases; close contacts were defined as unmasked persons who were within 6 ft of a person with a primary case for >15 minutes during a 24-hour period while that person was infectious, which was 2 days before through 10 days after symptom onset (for symptomatic persons) or after specimen collection date (for asymptomatic persons). Secondary cases were defined as COVID-19 cases that occurred in close contacts of persons with a primary case of confirmed or probable COVID-19.

Persons who had received 2 doses of Pfizer BioNTech or Moderna COVID-19 mRNA vaccine or 1 dose of Janssen (Johnson & Johnson) COVID-19 vaccine ≥14 days before exposure were considered fully vaccinated. IDPH laboratories performed whole genome sequencing on 25 of 31 available specimens.^¶^ Descriptive analyses were conducted to determine the distribution of cases by vaccination status, the proportion of SARS-CoV-2 variants, and the secondary transmission rate. This activity was reviewed by CDC and was conducted consistent with applicable federal law and CDC policy.**

As of August 13, a total of 180 outbreak-associated cases had been identified, including 122 primary cases, 87 (48%) of which were in camp attendees (among 335 total campers and staff members; attack rate = 26%) and 35 (19%) in conference attendees (among 530 total conference participants and staff members; attack rate = 7%). Among 262 close contacts of camp or conference attendees, 58 (22%) secondary cases were identified, representing 32% of the 180 identified cases (Figure) (Table). Among the 87 persons with camp-associated cases, none reported symptom onset before the camp started on June 13. Among the 35 persons with conference-associated cases, three reported being symptomatic during the conference (not including one camp-associated staff member who attended the conference while symptomatic).

**FIGURE:**
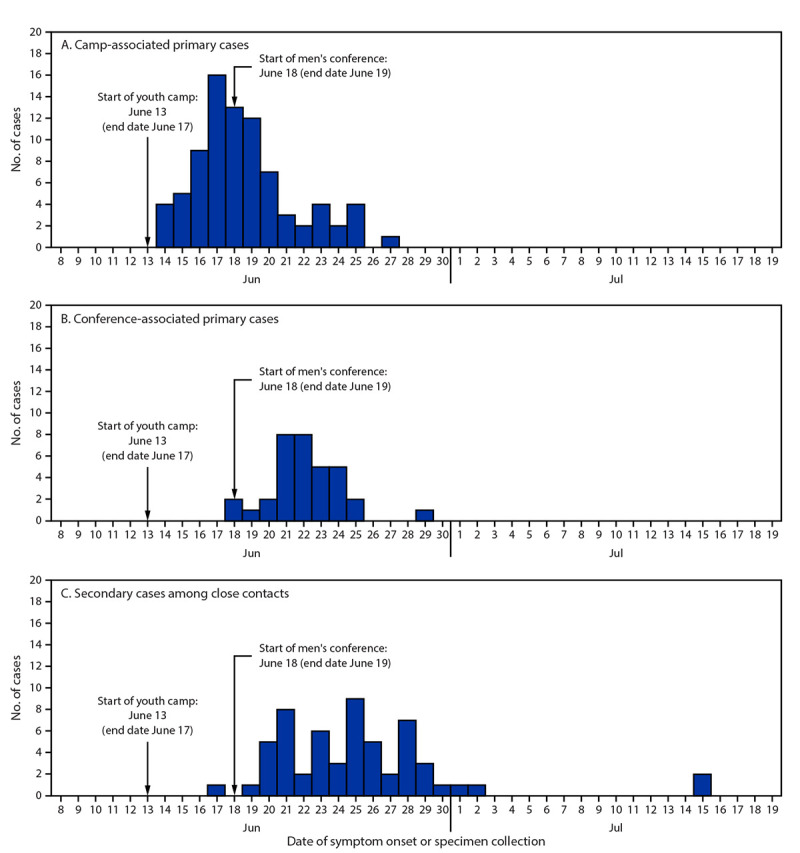
Number of primary COVID-19 cases among attendees of a youth camp (A) and men’s conference (B) and secondary cases among close contacts* (C), by date of symptom onset or specimen collection^†^ — Illinois, June–July 2021 * Close contacts were defined as unmasked persons who were within 6 ft of a person with a camp- or conference-associated (primary) case for >15 minutes during a 24-hour period while that person was infectious (i.e., 2 days before through 10 days after symptom onset or specimen collection date). Secondary cases were defined as COVID-19 cases in close contacts of persons with a primary case of confirmed or probable COVID-19 (confirmed: receipt of a positive SARS-CoV-2 nucleic acid amplification test result in an attendee; probable: receipt of a positive SARS-CoV-2 antigen test result). ^†^ Symptom onset date or specimen collection date was missing for six out-of-state persons. Among the remaining 174, onset date was available for 158 (91%), and specimen collection date was available for 16 (9%).

**TABLE:** Characteristics of persons with primary COVID-19 cases after attendance at a youth camp or men’s conference and of close contacts with secondary COVID-19 cases — Illinois, June–July 2021

Characteristic	No. (%)			
	Camp cases	Conference cases	Secondary cases	Total
**Minimum no. of persons exposed**	**335**	**530**	**262**	**1,127**
Reported cases*	87 (26)	35 (7)	58 (22)	**180 (16)**
**Median age, yrs (range)^†^**	17 (13–54)	44 (15–68)	38 (3–72)	**26 (3–72)**
**Sex^†^**				
Male	28 (34)	35 (100)	20 (34)	**83 (47)**
Female	55 (66)	0 (—)	38 (66)	**93 (53)**
**Persons who required emergency department care**	3 (3)	5 (14)	5 (9)	**13 (7)**
**Persons hospitalized**	1 (1)	3 (9)	1 (2)	**5 (3)**
**Fully vaccinated persons^§^**	8 (9)	10 (29)	11 (19)	**29 (16)**
**Vaccine product received by fully vaccinated persons**				
Pfizer-BioNTech	3 (38)	5 (50)	3 (27)	**11 (41)**
Moderna	2 (25)	3 (30)	2 (18)	**7 (24)**
Janssen (Johnson & Johnson)	3 (38)	2 (20)	6 (55)	**11 (38)**
**Unknown vaccine product received or partially vaccinated**	79 (91)	25 (71)	47 (81)	**151 (84)**
**No. of viruses sequenced^¶^**	15	8	8	**31**
B.1.617.2 (Delta)	13 (87)	7 (88)	7 (88)	**27 (87)**
B.1.1.7 (Alpha)	1 (7)	1 (13)	1 (13)	**3 (10)**
P.1 (Gamma)	1 (7)	0 (—)	0 (—)	**1 (3)**
Minimum no. of affected counties**	16	7	8	**18**

* Percentages might not sum to 100% because of rounding.

^†^ For 174 of 180 cases in persons with a reported onset or specimen collection date, sex (176), and date of birth.

^§^ Fully vaccinated persons were defined as persons who had received 2 doses of Pfizer BioNTech or Moderna COVID-19 mRNA vaccine or 1 dose of Janssen (Johnson & Johnson) COVID-19 vaccine ≥14 days before exposure.

^¶^ Illinois Department of Public Health laboratories performed whole genome sequencing on 25 of 31 specimens using Illumina’s NextSeq 550 instrument with the COVID-Seq workflow. Consensus genome assembly was performed in Illumina’s DRAGEN analytical pipeline, and variants were assigned using the most recent Pangolin version.

** Because seven of 16 counties of residence identified among camp and conference attendees were outside of Illinois, contact tracing data were extremely limited.

Among the 180 total persons with outbreak-associated cases, 13 (7.2%) required medical care in an emergency department, and five (2.8%) were hospitalized; no deaths occurred (Table). None of the vaccinated persons with cases were hospitalized; three sought care at an emergency department. Overall, 29 (16.1%) cases occurred in fully vaccinated persons (camp cases: 9%; conference cases: 29%). Among the 262 close contacts of persons with a primary case, 52 (20%) were fully vaccinated; 11 of these fully vaccinated persons received a positive SARS-CoV-2 test result, representing 19% of the 58 secondary cases among close contacts of camp or conference attendees with primary cases.

Among the 122 cases in camp or conference attendees, 18 were in fully vaccinated persons (eight in camp attendees and 10 in conference attendees). These 18 fully vaccinated persons reported a total of 38 close contacts; eight (21%) of these close contacts received positive SARS-CoV-2 test results, four (50%) of whom were fully vaccinated. Among the 224 reported close contacts of unvaccinated and partially vaccinated persons with primary cases, 50 (22%) received positive SARS-CoV-2 test results, including seven fully vaccinated persons. Among 58 persons with secondary cases, 48 (83%) were infected by household members, four by nonhousehold family members, three by friends, and one each by a neighbor, at work, or during a Bible study group.

Overall, 1,127 persons from at least four states and 18 counties were exposed to SARS-CoV-2 through attendance at the camp or conference or through close contact with a person who had a camp- or conference-associated case. In the 7 days before the camp (June 6–12), Adams County, Illinois,^††^ reported 31 COVID-19 cases, with an average of 4.4 cases per day. In the 7 days after the last identified secondary case (July 16–22), the county reported 232 cases, with an average of 33.1 per day, a 648% increase from the number reported during the week before the camp (*2*).

Among samples sequenced from specimens from 31 infected persons (15 from camp-associated cases, eight from conference-associated cases, and eight from secondary cases), the B.1.617.2 (Delta) variant was identified in 27 (87%), including two AY.3 (Delta) sequences; the B.1.1.7 (Alpha) variant was identified in three (10%); and the P.1 (Gamma) variant was identified in one (3%) (Table). Among eight sequenced samples from specimens from vaccinated persons, the Delta variant was identified in seven samples, and Alpha in one.^§§^

## Public Health Response

IDPH sent three Epi-X notifications^¶¶^ about these outbreaks to state and local health departments and received case data from the state health departments in Iowa, Michigan, and Missouri. On June 30, IDPH requested CDC’s assistance with investigating these outbreaks. On July 19, a CDC field team arrived in Illinois to assist with active case finding in several jurisdictions, collection and analysis of samples, and ascertainment of secondary transmission. As of August 13, complete rosters of attendees and staff members at both events were not available.

## Discussion

COVID-19 vaccines currently authorized by the Food and Drug Administration are safe and highly effective for preventing COVID-19–related serious illness, hospitalization, and death.*** In this investigation, most reported COVID-19 cases were identified among unvaccinated persons. However, transmission of SARS-CoV-2 from vaccinated persons both to unvaccinated and vaccinated persons likely occurred. These breakthrough cases among vaccinated persons were identified among attendees of the camp and the conference and in persons exposed to the attendees. Consistent with previous studies, much of the identified secondary transmission occurred within households, where most prolonged contact occurs (*3*).

Approximately 1,000 persons in at least four states were exposed to SARS-CoV-2 through attendance at the camp or conference or through close contact with a person infected at the event. The high rate of transmission was likely driven by the number of persons infected with the SARS-CoV-2 Delta variant. However, because multiple SARS-CoV-2 variants of concern were identified from the specimens of camp attendees, this suggests multiple introductions of SARS-CoV-2 into the camp, rather than a single introduction event. As of August 7, COVID-19 outbreaks in at least 21 overnight camps had been reported in Illinois, reinforcing the importance of COVID-19 prevention measures at these camps, including identifying infected persons through prearrival and screening testing programs and consistent implementation of other prevention efforts, including vaccination, masking, and physical distancing (*4*–*6*). Several camp staff members who were infected with SARS-CoV-2 (including at least one symptomatic person) or who had been exposed to the virus attended another large group event during their infectious period. Therefore, timely notification of all close contacts and compliance with isolation and quarantine guidance are also critical. 

The findings in this report are subject to at least two limitations. First, the investigation likely underestimates the number of SARS-CoV-2 primary infections, secondary exposures, and secondary cases because the case definition required laboratory confirmation; therefore, infected persons who did not receive testing or who used at-home SARS-CoV-2 antigen tests (i.e., self-collection kits) were not included in the case count. In addition, not all persons with cases participated in contact tracing; the close contacts of persons who did participate were likely underreported and were biased toward household contacts (*7*). Second, investigators did not have access to complete rosters for the camp or conference, which limited case finding efforts and analyses involving persons who were not infected (particularly findings related to vaccination status).

These findings underscore the risk for COVID-19 outbreaks at camps and large events where prevention strategies are not implemented and highlight the importance of implementing such strategies to reduce transmission of SARS-CoV-2 in these settings (*8*). Promoting vaccination, implementing and encouraging compliance with prompt quarantine and isolation measures for exposed and infected persons, staying home when sick, and using nonpharmaceutical interventions including masking, physical distancing, and screening testing in large group settings can help reduce secondary infections in homes and the community and serious complications from COVID-19 (*9*).

**Summary**

**What is already known about this topic?**

The Illinois Department of Public Health investigated COVID-19 outbreaks at two events sponsored by the same organization: a 5-day overnight church camp for persons aged 14–18 years and a 2-day men’s conference.

**What is added by this report?**

Neither COVID-19 vaccination nor COVID-19 testing was required before either event. Among 122 primary cases, 104 (85%) were in persons who were not fully vaccinated, and 18 (15%) were in fully vaccinated persons. Eight of 38 (21%) close contacts of the 18 fully vaccinated persons subsequently became infected with SARS-CoV-2. No vaccinated persons with COVID-19 were hospitalized.

**What are the implications for public health practice?**

This investigation underscores the impact of secondary SARS-CoV-2 transmission during large events such as camps and conferences when COVID-19 prevention strategies, including vaccination, masking, physical distancing, and screening testing, are not implemented.

## Footnotes

* Persons with positive SARS-CoV-2 test results were instructed to isolate in accordance with CDC guidance at the time (https://www.cdc.gov/coronavirus/2019-ncov/hcp/duration-isolation.html), and close contacts were instructed to quarantine in accordance with CDC guidance at the time (https://www.cdc.gov/coronavirus/2019-ncov/if-you-are-sick/quarantine.html).

† Six staff members (three at the camp and three at the conference) were identified as having cases on the basis of their symptom onset date. Because the complete rosters had not been provided to IDPH, persons might have been counted as attendees at both events.

§
https://ndc.services.cdc.gov/case-definitions/coronavirus-disease-2019-2020-08-05/

¶ Whole genome sequencing was performed on Illumina’s NextSeq 550 instrument using the COVID-Seq workflow. Consensus genome assembly was performed in Illumina’s DRAGEN analytical pipeline, and variants were assigned using the most recent Pangolin version.

** 45 C.F.R. part 46.102(l)(2), 21 C.F.R. part 56; 42 U.S.C. Sect. 241(d); 5 U.S.C. Sect. 552a; 44 U.S.C. Sect. 3501 et seq.

†† Adams County is used as the reference county because it was the county of residence for 49% (43) of persons with camp-associated cases, 40% (14) of persons with conference-associated cases, and 59% (34) of persons with secondary cases.

§§ Six of these eight vaccinated persons who were infected with SARS-CoV-2 variants of concern were from households with two persons with sequenced samples; two pairs of the Delta variant and one discordant pair of Delta and Alpha variants were identified in samples from these households.

¶¶ Epi-X is a secure, web-based notification system, with approximately 6,000 users from public health agencies, that guides and coordinates public health professionals during public health threats and investigations. https://emergency.cdc.gov/epix/index.asp

***
https://www.cdc.gov/coronavirus/2019-ncov/vaccines/vaccine-benefits.html
